# Human Mesenchymal Stromal Cells Do Not Cause Radioprotection of Head-and-Neck Squamous Cell Carcinoma

**DOI:** 10.3390/ijms23147689

**Published:** 2022-07-12

**Authors:** Alexander Rühle, Marie Lies, Maren Strack, Ramon Lopez Perez, Birgit Bieber, Andreas R. Thomsen, Peter Bronsert, Peter E. Huber, Jochen Hess, Andreas Knopf, Patrick Wuchter, Anca-Ligia Grosu, Nils H. Nicolay

**Affiliations:** 1 Department of Radiation Oncology, Medical Center, Faculty of Medicine, University of Freiburg, Robert-Koch-Str. 3, 79106 Freiburg, Germany; alexander.ruehle@uniklinik-freiburg.de (A.R.); marie.lies@uniklinik-freiburg.de (M.L.); maren.strack@uniklinik-freiburg.de (M.S.); birgit.bieber@uniklinik-freiburg.de (B.B.); andreas.thomsen@uniklinik-freiburg.de (A.R.T.); anca.grosu@uniklinik-freiburg.de (A.-L.G.); 2 German Cancer Consortium (DKTK) Partner Site Freiburg, German Cancer Research Center (DKFZ), 69120 Heidelberg, Germany; 3 Department of Molecular and Radiooncology, German Cancer Research Center (DKFZ), 69120 Heidelberg, Germany; r.lopez@dkfz-heidelberg.de (R.L.P.); p.huber@dkfz.de (P.E.H.); 4 Institute for Surgical Pathology, Medical Center, Faculty of Medicine, University of Freiburg, 79106 Freiburg, Germany; peter.bronsert@uniklinik-freiburg.de; 5 Core Facility for Histopathology and Digital Pathology, Medical Center, Faculty of Medicine, University of Freiburg, 79106 Freiburg, Germany; 6 Research Group Molecular Mechanisms of Head and Neck Tumors, German Cancer Research Center (DKFZ), 69120 Heidelberg, Germany; j.hess@dkfz.de; 7 Section Experimental and Translational Head and Neck Oncology, Department of Otolaryngology, Head and Neck Surgery, University Hospital Heidelberg, 69120 Heidelberg, Germany; 8 Department of Otorhinolaryngology, Medical Center, Faculty of Medicine, University of Freiburg, 79106 Freiburg, Germany; andreas.knopf@uniklinik-freiburg.de; 9 Institute of Transfusion Medicine and Immunology, Medical Faculty Mannheim, Heidelberg University, German Red Cross Blood Service Baden-Württemberg-Hessen, 68167 Mannheim, Germany; patrick.wuchter@medma.uni-heidelberg.de

**Keywords:** mesenchymal stromal cells, head-and-neck squamous cell carcinoma, head-and-neck cancer, radiotherapy, radiosensitivity, radioresistance, co-culture

## Abstract

Radiotherapy of head-and-neck squamous cell carcinoma (HNSCC) can cause considerable normal tissue injuries, and mesenchymal stromal cells (MSCs) have been shown to aid regeneration of irradiation-damaged normal tissues. However, utilization of MSC-based treatments for HNSCC patients undergoing radiotherapy is hampered by concerns regarding potential radioprotective effects. We therefore investigated the influence of MSCs on the radiosensitivity of HNSCCs. Several human papillomavirus (HPV)-negative and HPV-positive HNSCCs were co-cultured with human bone marrow-derived MSCs using two-dimensional and three-dimensional assays. Clonogenic survival, proliferation, and viability of HNSCCs after radiotherapy were assessed depending on MSC co-culture. Flow cytometry analyses were conducted to examine the influence of MSCs on irradiation-induced cell cycle distribution and apoptosis induction in HNSCCs. Immunofluorescence stainings of γH2AX were conducted to determine the levels of residual irradiation-induced DNA double-strand breaks. Levels of connective tissue growth factor (CTGF), a multifunctional pro-tumorigenic cytokine, were analyzed using enzyme-linked immunosorbent assays. Neither direct MSC co-culture nor MSC-conditioned medium exerted radioprotective effects on HNSCCs as determined by clonogenic survival, proliferation, and viability assays. Consistently, three-dimensional microwell arrays revealed no radioprotective effects of MSCs. Irradiation resulted in a G2/M arrest of HNSCCs at 96 h independently of MSC co-culture. HNSCCs’ apoptosis rates were increased by irradiation irrespective of MSCs. Numbers of residual γH2AX foci after irradiation with 2 or 8 Gy were comparable between mono- and co-cultures. MSC mono-cultures and HNSCC-MSC co-cultures exhibited comparable CTGF levels. We did not detect radioprotective effects of human MSCs on HNSCCs. Our results suggest that the usage of MSC-based therapies for radiotherapy-related toxicities in HNSCC patients may be safe in the context of absent radioprotection.

## 1. Introduction

Head-and-neck squamous cell carcinoma (HNSCC) is a common cancer type, leading to high morbidity and mortality worldwide [1]. Radiotherapy is a therapeutic mainstay for HNSCCs both as a primary treatment modality and as an adjuvant therapy after surgical procedures [2,3,4]. Nevertheless, radiotherapy can cause considerable injuries to normal tissues in the head-and-neck region, leading to both acute (e.g., mucositis, dermatitis) and chronic sequelae (e.g., osteoradionecrosis, xerostomia) [5,6,7]. Patient outcomes after radiotherapy have improved over the last decades, and especially patients with human papillomavirus (HPV)-positive oropharynx cancers exhibit a high chance of long-term survival [8,9,10,11]. Better prognoses result in an increasing need for causative treatments of chronic toxicities in order to improve patients’ long-term quality-of-life. In this context, several preclinical and some clinical studies have shown the potential of stem cell-based therapies for radiotherapy-induced normal tissue toxicities [12,13,14,15].

Mesenchymal stromal cells (MSCs) are multipotent cells that were first isolated from bone marrow samples but can be found in many different tissues [16,17,18]. Due to their regenerative abilities, MSC-based treatments have been widely investigated for the attenuation of radiotherapy- and chemotherapy-related normal tissue toxicities [19,20,21,22,23]. The ability of MSCs to migrate to irradiated tissues and to aid regeneration both by producing a protective microenvironment and by differentiating into functional tissue-specific cells forms the rationale to study MSCs as potential attenuators of radiotherapy-related adverse reactions [19,24,25]. However, MSCs have been suspected to exhibit pro-tumorigenic and radioprotective effects, although this issue remains controversial, as these abilities seem to be highly dependent on the MSC source, tumor type, and tumor microenvironment [26,27]. Therefore, MSC-based treatments for cancer patients suffering from radiotherapy-induced toxicities are still controversially discussed. Only a few studies have analyzed the interaction between MSCs and HNSCCs in the context of cancer progression, hypoxia, and drug resistance, and there is to the best of our knowledge no study in which the risk for MSC-mediated radioprotection of HNSCCs has been investigated [28,29,30].

We therefore aimed to explore the interaction between MSCs and HNSSCs regarding tumor radioprotection in a comprehensive in vitro study using both two- and three-dimensional models.

## 2. Results

### 2.1. Neither Direct nor Indirect MSC Co-Culture Result in Increased Radioresistance of HNSCCs

**Figure 1:**
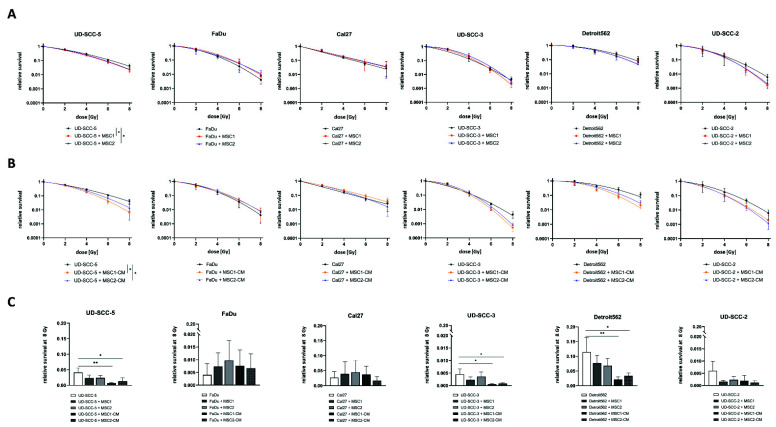
**Neither direct nor indirect MSC co-culture increase HNSCCs’ radioresistance.** (**A**,**B**) Clonogenic survival assays of HNSCCs either alone, co-cultured with MSCs (**A**) or co-cultured in MSC-CM (**B**) after irradiation. Clonogenic survival curves were modeled using the linear-quadratic model. * *p* < 0.05 (paired *t*-test). (**C**) Relative survival rates at 8 Gy for HNSCC mono-culture, direct HNSCC-MSC co-culture, and indirect (i.e., MSC-CM) HNSCC-MSC co-culture. Mean values with the according standard deviations are shown. Here, each group was compared with the HNSCC mono-culture using a one-way ANOVA with post-hoc Dunnett’s tests. * *p* < 0.05, ** *p* < 0.01.

Neither culturing HNSCCs with MSCs nor in MSC-CM resulted in a radioprotection of any of the tumor cells (Figure 1). On the contrary, both direct MSC co-culturing and MSC-CM significantly increased sensitivity of UD-SCC-5 cells to radiation treatment (*p* < 0.05, paired *t*-test). Sensitizer enhancement ratio (SER) values amounted to 1.13 and 1.10 for MSC1 and MSC2, respectively, and 1.28 and 1.17 for MSC1-CM and MSC2-CM, respectively (Table S1). After irradiation with 8 Gy, CM of both MSC1 (*p* < 0.01, ANOVA test) and MSC2 (*p* < 0.05) impaired clonogenic survival by the factor 5.9 (MSC1-CM) and 3.1 (MSC2-CM) compared to the UD-SCC-5 monoculture. Similarly, the HPV-positive HNSCC cell line UD-SCC-2 exhibited a trend towards reduced clonogenic survival after co-culture with MSC-CM, although statistical significance was not reached (*p* = 0.12 for MSC1, *p* = 0.06 for MSC2). Here, the SER values were 1.19 for MSC1-CM and 1.21 for MSC2-CM.

Direct MSC co-culturing did not alter the baseline plating efficiency of any tested HNSCC cell line in the clonogenic survival assays, whereas MSC-CM increased the plating efficiency at 0 Gy for UD-SCC-3 (*p* < 0.01 for MSC1-CM and MSC2-CM), Detroit562 (*p* < 0.05 for MSC1-CM, *p* < 0.01 for MSC2-CM), and UD-SCC-2 (*p* < 0.01 for MSC1-CM) (Figure S1).

**Figure 2:**
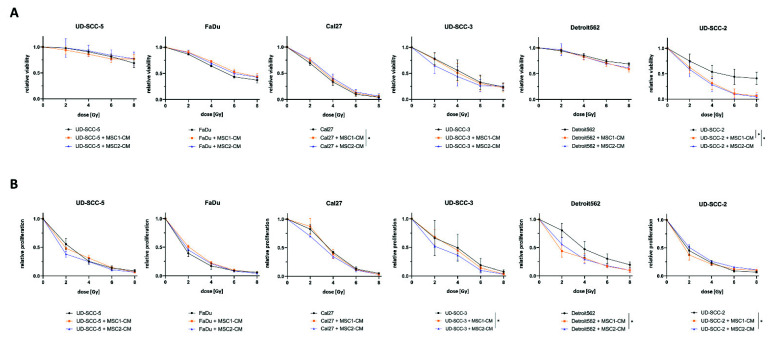
**MSC-CM does not increase viability or cellular proliferation in HNSCCs after irradiation.** (**A**) Relative viability measured by resazurin assays of different HNSCC cell lines at 96 h after irradiation depending on MSC-CM. (**B**) Relative proliferation of HNSCCs at 96 h after irradiation determined by trypan blue stainings. HNSCCs were either cultured in HNSCC medium or in MSC-CM. * *p* < 0.05 (paired *t*-test).

Cellular viability as measured by resazurin assays revealed a radiosensitizing impact of MSC1-CM (*p* < 0.05) and MSC2-CM (*p* < 0.05) on the HPV-positive HNSCC cell line UD-SCC-2: Viability rates were only reduced by 59.0% after 8 Gy compared to untreated controls in the mono-culture, whereas it was reduced by 93.6% (MSC1-CM) and 95.8% (MSC2-CM) at the same irradiation dose when cultured in MSC-CM (Figure 2). Cal27 cells showed a marginal but statistically significant increase of their cellular viability after co-culturing in MSC2-CM (*p* < 0.05), whereas there was no impact of MSC-CM on irradiation-induced viability decrease for the other HPV-negative HNSCCs.

Irradiation-induced reduction of cellular proliferation was found pronounced for UD-SCC-3 (*p* < 0.05) when co-cultured in MSC2-CM. UD-SCC-2 cells co-cultured in MSC2-CM showed a slightly increased proliferation after irradiation compared to UD-SCC-2 mono-cultures (*p* < 0.05). The other HNSCC cell lines exhibited no differences between the mono-culture and the indirect HNSCC-MSC co-culture in terms of proliferation after exposure to ionizing radiation.

The basal proliferation rate was found enhanced in presence of MSC-CM for FaDu (*p* < 0.01 for MSC1-CM and MSC2-CM), UD-SCC-3 (*p* < 0.05 for MSC1-CM, *p* < 0.001 for MSC2-CM), Detroit562 (*p* < 0.001 for MSC1-CM, *p* < 0.01 for MSC2-CM), and UD-SCC-2 (*p* < 0.05 for MSC1-CM), whereas there was no effect on UD-SCC-5 and Cal27 (Figure S2). Following the results of the clonogenic survival assay, the effect was most pronounced for UD-SCC-3.

### 2.2. Direct Three-Dimensional MSC-HNSCC Co-Culture Reveals No Radioprotective Effects of MSCs

**Figure 3:**
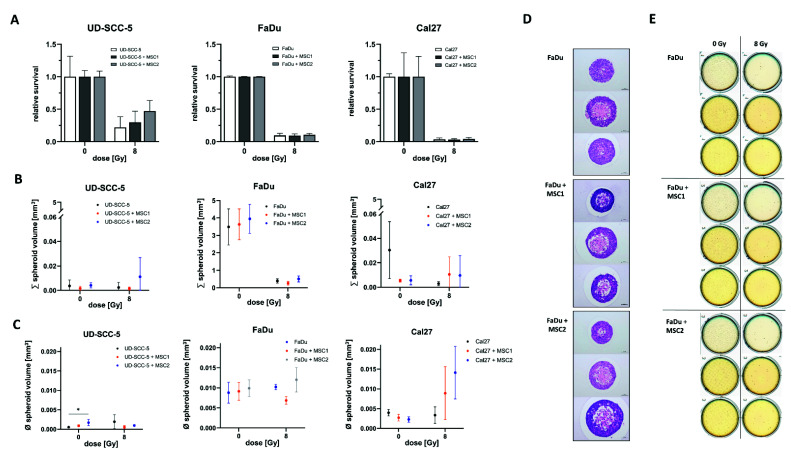
**Direct three-dimensional MSC-HNSCC co-culture reveals no radioprotective effects of MSCs.** (**A**) Relative survival after irradiation with 8 Gy in HNSCCs mono-culture and HNSCC-MSC co-culture. By comparing HNSCC mono-cultures with HNSCC-MSC co-cultures using one-way ANOVA with post-hoc Dunnett’s tests, no radioprotective or radiosensitizing effects could be observed. (**B**) Sum of all spheroids within the conical agarose microwell arrays at 0 Gy and 8 Gy. One-way ANOVA tests revealed no differences between HNSCC mono-culture and HNSCC-MSC co-cultures. (**C**) Average spheroid volume of HNSCC mono-culture and HNSCC-MSC co-cultures after 0 Gy and 8 Gy. * *p* < 0.05 (one-way ANOVA with post-hoc Dunnett’s tests). (**D**) Representative images of hematoxylin and eosin stainings. (**E**) Representative scans of the HNSCC and HNSCC-MSC conical agarose microwell arrays after 0 Gy or 8 Gy, showing the anti-proliferative effects of 8 Gy irradiation on the cell aggregates.

In line with the survival data obtained from two-dimensional cell culture experiments, we could not detect any radioprotective effects of MSCs on HNSCCs in three-dimensional agarose microwell arrays (Figure 3). Neither MSC1 nor MSC2 significantly influenced the radiosensitivity of the analyzed HNSCC cell lines.

Furthermore, there were no differences regarding the aggregated spheroid volume between the HNSCC mono-culture and HNSCC-MSC co-cultures. Only UD-SCC-5 spheroids exhibited increased volumes in average when co-cultured with MSC2 (*p* < 0.05).

### 2.3. Irradiation-Induced G2/M Cell Cycle Arrest in HNSCCs Is Not Abrogated by MSCs

**Figure 4:**
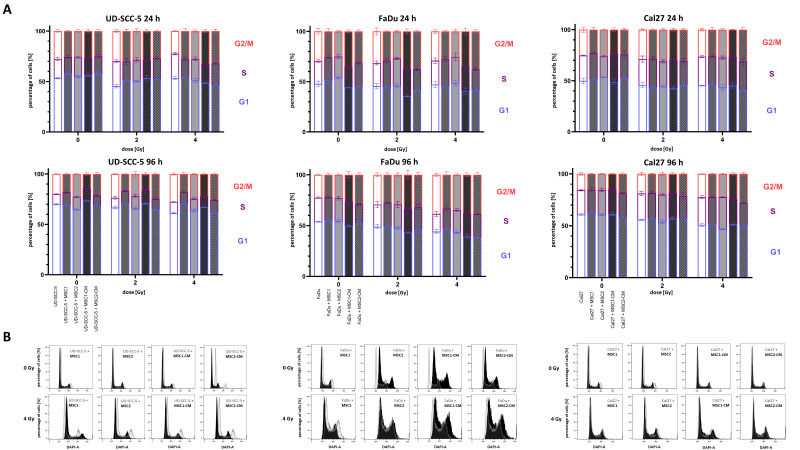
**Irradiation-induced G2/M arrest in HNSCCs it not abrogated by MSC co-culturing.** (**A**) Cell cycle distribution in HNSCCs at 24 h and 96 h depending on direct or indirect MSC co-culture. Statistics are shown in Figure S3. (**B**) Representative cell cycle histograms of HNSCC mono-culture (grey) or HNSCC-MSC co-culture (black).

4′,6-diamidino-2-phenylindole (DAPI) measurements were used to analyze the cell cycle distribution of HNSCCs after irradiation depending on MSC co-culture. At 96 h after irradiation, there was a dose-dependent increase of cells in the G2/M phase in all tested HNSCC cell lines (Figure 4 and Figure S3 ). While 19.8%, 22.5%, and 15.7% of UD-SCC-5, FaDu, and Cal27 cells were in the G2/M phase in the control experiments, respectively, it increased to 27.7%, 38.4%, and 22.6%, respectively, after irradiation with 4 Gy. In general, the irradiation-induced G2/M arrest in HNSCCs was present independent of direct or indirect MSC co-culture. However, there were some slight alterations regarding the amount of cells in the G2/M phase after irradiation: for instance, the irradiation-induced G2/M arrest was found weakened in the co-cultures of UD-SCC-5 and MSCs compared to the UD-SCC-5 mono-cultures at 96 h after irradiation (after 2 Gy: *p* < 0.001 for MSC1 co-culture, *p* < 0.001 for MSC1-CM; after 4 Gy: *p* < 0.001 for MSC1 co-culture, *p* < 0.05 for MSC2 co-culture, *p* < 0.01 for MSC1-CM).

### 2.4. MSCs Do Not Suppress HNSCC Apoptosis Rates after Radiation Treatment

**Figure 5:**
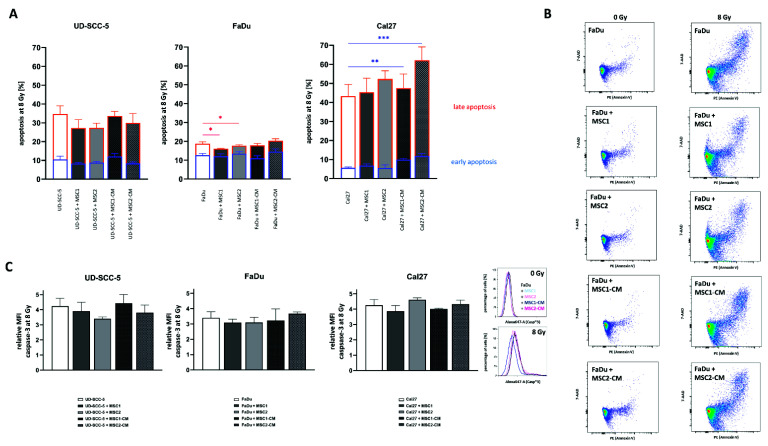
**HNSCCs’ apoptosis rates after irradiationare unaltered after MSC co-culture.** (**A**) Rates of irradiation-induced early (blue) and late (orange) apoptosis at 96 h in HNSCCs using annexin V/7-AAD stainings. (**B**) Representative flow cytometry plots of annexin V/7-AAD stainings in HNSCCs, either alone or co-cultured with MSCs or co-cultured in MSC-CM, after exposure to ionizing radiation. (**C**) Relative mean fluorescence intensity (MFI) of cleaved caspase-3 in irradiated HNSCCs depending on MSC co-culturing. Representative histograms show the fluorescence intensity of cleaved caspase-3 after irradiation in HNSCC mono-culture and in HNSCC-MSC co-cultures. Each HNSCC-MSC co-culture group was compared with the HNSCC mono-culture using one-way ANOVA with post-hoc Dunnett’s tests. * *p* < 0.05, ** *p* < 0.01, *** *p* < 0.001.

Flow cytometry analyses of both cleaved caspase-3 and annexin-V were carried out to analyze a potential impact of MSCs on the apoptosis levels of HNSCCs after irradiation with high irradiation doses (Figure 5). The mean fluorescence intensity of cleaved caspase-3 was increased more than three-fold in all tested HNSCCs at 96 h after 8 Gy. Irradiation-induced apoptosis rates were not abrogated or elevated when HNSCCs were co-cultured with MSCs or MSC-CM. In line with the results of the caspase-3 analyses, annexin-V/7-AAD experiments could not reveal protective effects of MSCs or MSC-CM regarding HNSCC apoptosis rates after irradiation. Irradiation with 8 Gy considerably increased both early and late apoptosis rates in HNSCCs. While early apoptosis rates (annexin-V-positive/7-AAD-negative) ranged below 5% in unirradiated controls (Figure S4), they doubled at 96 h after 8 Gy (UD-SCC-5: 10.5%, FaDu: 12.6%, Cal27: 5.6%). The increase in late apoptosis (annexin-V-positive/7-AAD-positive) was even more pronounced: Irradiation with 8 Gy resulted in 24.2% UD-SCC-5, 6.1% FaDu, and 37.7% Cal27 cells showing signs of late apoptosis. In general, MSC co-cultures did not influence the rates of early apoptosis in HNSCCs after irradiation. MSC-CM even increased the levels of irradiation-induced early apoptosis induction in Cal27 (*p* < 0.01 for MSC1-CM, *p* < 0.001 for MSC2-CM). Only in FaDu cells, there was a slight reduction of annexin-V-positive/7-AAD-positive cells in direct HNSCC-MSC co-cultures compared to the HNSCC mono-culture (*p* < 0.05).

### 2.5. Levels of Residual DNA Double-Strand Breaks after Irradiation Are Similar between HNSCC Mono-Cultures and HNSCC-MSC Co-Cultures

**Figure 6:**
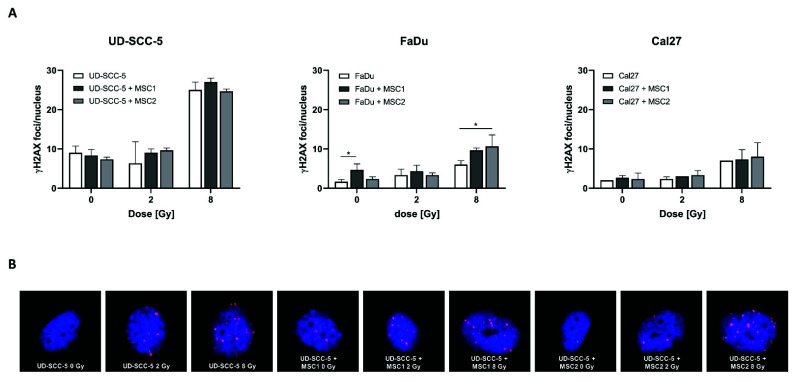
**Levels of residual γH2AX foci after irradiation are largely comparable between HNSCC mono-cultures and HNSCC-MSC co-cultures.** (**A**) γH2AX foci/nucleus in UD-SCC-5, FaDu, and Cal27 cells, either in mono-culture or in co-cultures with MSCs, at 24 h after sham-irradiation or irradiation with 2 Gy or 8 Gy. (**B**) Representative images of UD-SCC-5 cells after sham-irradiation or irradiation with 2 Gy or 8 Gy. For each irradiation dose, the HNSCC-MSC co-culture group was compared with the HNSCC mono-culture using one-way ANOVA with post-hoc Dunnett’s tests. * *p* < 0.05.

γH2AX immunofluorescence stainings revealed an increase of DNA double-strand breaks at 24 h after irradiation with 8 Gy compared to unirradiated control samples (Figure 6). In contrast, all three analyzed HNSCC cell lines (UD-SCC-5, FaDu, Cal27) exhibited similar numbers of γH2AX foci per nucleus after 2 Gy irradiation when compared with sham-irradiated controls. While there was no difference in the number of residual DNA double-strand breaks between the HNSCC mono-culture and the HNSCC-MSC co-culture in UD-SCC-5 and Cal27 cells, there was a significant increase of residual γH2AX foci levels in FaDu cells co-cultured with MSCs: While there were less than 2 γH2AX foci per nucleus in unirradiated FaDu cells, the average foci number was about 5 in unirradiated FaDu-MSC1 co-cultures (*p* < 0.05). There was no difference in terms of γH2AX foci/nucleus between FaDu mono-cultures and FaDu-MSC co-cultures after 2 Gy irradiation; however, there was a significant increase of irradiation-induced γH2AX foci levels in FaDu cells when co-cultured with MSCs after 8 Gy (*p* < 0.05 for MSC2, *p* = 0.08 for MSC1).

### 2.6. CTGF Secretion Is Increased in HNSCC-MSC Co-Cultures Compared to HNSCC Mono-Cultures but Comparable to MSC Mono-Cultures

**Figure 7:**
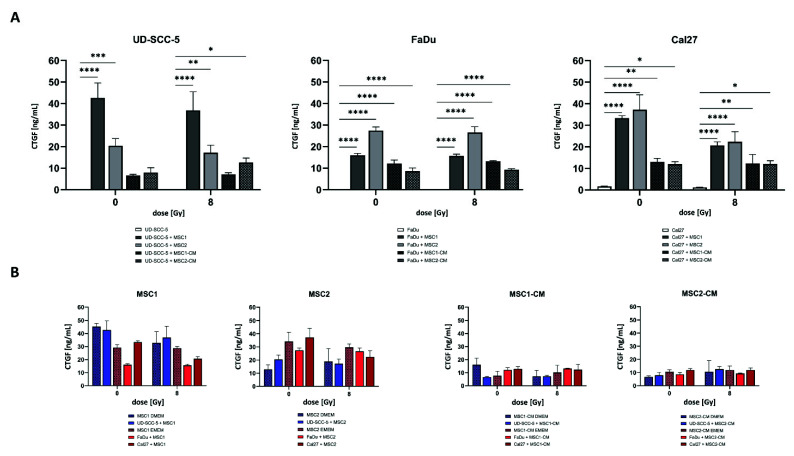
CTGF secretion is increased in HNSCC-MSC co-cultures compared to HNSCC mono-cultures but comparable to MSC mono-cultures. (**A**) CTGF levels after irradiation in HNSCC mono-cultures and HNSCC-MSC co-cultures determined by ELISA measurements. Comparisons between HNSCC mono-culture and HNSCC-MSC co-culture were carried out using one-way ANOVA with post-hoc Dunnett’s tests. (**B**) CTGF levels in MSCs mono-culture and direct HNSCC-MSC co-cultures (left side). CTGF levels in MSC-CM (i.e., without exposure to HNSCCs) and indirect HNSCC-MSC co-cultures (i.e., HNSCCs cultured in MSC-CM) (right side). Here, CTGF levels in MSCs were compared with levels in direct HNSCC-MSC co-cultures (left side), or between MSC-CM and HNSCCs cultured in MSC-CM (right side) by using one-way ANOVA with post-hoc Dunnett’s tests. In order to take the different cell culture media into account, only groups with the same medium were compared (blue and red bars were compared separately). * *p* < 0.05, ** *p* < 0.01, *** *p* < 0.001, **** *p* < 0.0001.

CTGF as a critical protein involved in biological processes of HNSCC such as cancer progression and mesenchymal-epithelial transition was investigated using enzyme-linked immunosorbent assay (ELISA) measurements. While there were no relevant CTGF levels in HNSCC mono-cultures, CTGF concentrations were found considerably enhanced when tumor cells were co-cultured with MSCs without treatment (*p* < 0.001 for UD-SCC-5-MSC2 co-culture, *p* < 0.0001 for all other comparisons) and after 8 Gy irradiation (*p* < 0.01 for UD-SCC-5-MSC2 co-culture, *p* < 0.0001 for all other comparisons) (Figure 7). Importantly, CTGF levels in MSC-CM did not increase after culturing of HNSCCs, and MSC mono-cultures exhibited similar CTGF levels as HNSCC-MSC co-cultures (also recognizing that two types of cell culture media were used), suggesting that CTGF levels were almost exclusively and constitutively secreted by MSCs, and the presence of HNSCCs did not stimulate CTGF secretion.

## 3. Discussion

In this comprehensive preclinical study, we could demonstrate that MSCs do not increase HNSCCs’ radioresistance in vitro. Apoptosis rates and levels of residual DNA double-strand breaks were found comparable between HNSCC mono-cultures and HNSCC-MSC co-cultures. Our results form a basis to further investigate MSCs as cell-based therapies for the treatment of radiotherapy-induced normal tissue toxicities in the head-and-neck region. Furthermore, our data do not support concerns about the application of MSC-based therapies in HNSCC patients undergoing radiotherapy.

Whereas we could not observe radioprotective abilities of MSCs on HNSCCs, there was at least one HNSCC cell line (UD-SCC-5, derived from a laryngeal carcinoma) that exhibited increased radiosensitivity in the clonogenic survival assays after co-culturing with either MSCs or MSC-CM. Other groups reported radiosensitizing effects of MSC-CM on breast cancer cells [31,32]. In another study, the CM of irradiated MSCs was found to inhibit melanoma cell growth, and combining radiotherapy and MSC administration resulted in synergistic cytotoxic effects on tumor cells in vitro and in vivo [33]. The same research group also discovered an anti-tumorigenic role of irradiation-induced MSC-derived exosomes on distant melanoma metastases, therefore potentially promoting abscopal effects after radiotherapy [34]. In an immunocompetent animal model of colon cancer treated by radiotherapy, bone marrow-MSCs impaired cancer in vivo by modulating the anti-tumor capacity of the immune system [35]. In this study, reduced chronic radiation-induced tissue toxicities such as fibrosis were observed in MSC-treated animals. However, in other tumor histologies, such as glioblastoma, the presence of tumor-associated MSCs was linked to diminished survival after surgery and radiotherapy [36]. Preliminary results of our group could demonstrate radioprotective effects of MSCs on primary glioblastoma cells, that would at least partly explain the negative prognostic role of tumor-associated MSCs in glioblastoma patients [37].

Regarding normal tissue protection, preclinical and clinical studies have suggested beneficial effects of MSC transplantation on salivary flow rates in xerostomia patients [13,38]. The randomized placebo-controlled MESRIX trial has successfully demonstrated both feasibility and efficacy of autologous MSC transplantation into submandibular glands of HNSCC patients suffering from irradiation-induced xerostomia [13]. In this trial, salivary flow rates significantly increased after autologous transplantation of adipose tissue-derived MSCs, resulting in reduced xerostomia and improved quality-of-life. Following these promising results, further studies including a long-term follow-up study of the MESRIX trial aim to analyze MSC-based applications as a potential therapy for radiotherapy-related xerostomia (NCT04489732, NCT03876197) [39]. Similar to our study in which MSCs harvested from the bone marrow were used, the MARSH study aims to utilize autologous bone marrow-derived IFNγ-stimulated MSCs as a therapeutic agent [39]. In line with the MESRIX trial, only patients without evidence of active tumor disease and at least two years away from completion of radiotherapy will be eligible for enrolment. Given the high prevalence of xerostomia after (chemo)radiotherapy in the head-and-neck region and the early onset after treatment [5], one may ask whether concomitant MSC autotransplantation during treatment would be more effective than post-therapeutic transplantation. In this context, systemic intravenous MSC (or MSC-CM) administration during radiotherapy could then also ameliorate radiation-induced mucositis and dermatitis considering the preclinical evidence [12,40,41,42,43,44]. While our data cannot fully rule out any pro-tumorigenic effects of MSCs in HNSCC patients, we could not demonstrate any radioprotective effects in longer-term three-dimensional co-culture models mimicking the clinical scenario, wherefore further studies with simultaneous MSC administration during radiotherapy seem worth considering, as far as thorough measures are followed to compare tumor recurrence rates between MSC treatment and control groups.

MSC-derived exosomes have shown to reduce radiotherapy-related normal tissue toxicities, for instance in the bone marrow [26,45]. In a mouse model, MSC-CM of MSCs cultured in hypoxia was demonstrated to improve salivation capacity after radiotherapy. Molecularly, MSC-CM strongly increased proliferation of human parotid epithelial cells and decreased irradiation-induced cell death rates in vitro and in vivo [46]. Furthermore, for other radiotherapy-related injuries in the head-and-neck region, such as mucositis and osteoradionecrosis, both cellular MSC therapies and MSC-CM have been studied [14,47]. We investigated both direct MSC co-culture and indirect MSC-CM co-culture and could not reveal radioprotective effects in any of the two approaches.

Importantly, MSCs derived from the bone marrow of healthy donors were used for the experiments, and we did not analyze whether tumor-derived MSCs could exert radioprotective effects on HNSCCs. Liotta and colleagues isolated HNSCC-derived MSCs and demonstrated strong immunosuppressive functions of these cells on lymphocytes, thereby negatively influencing the anti-tumor effect of tumor-infiltrating lymphocytes [48]. There are further studies that examined the interaction between MSCs and HNSCCs [49,50,51,52,53,54]. While we observed no or only minor alterations of the HNSCCs’ baseline apoptosis levels in dependence of MSC co-cultures, another study reported growth-inhibiting effects of tonsil-derived MSCs on HNSCCs [55]. In contrast, a recent study found enhanced proliferation rates and an increased invasive migration potential in HNSCCs after exposure to supernatant of adipose tissue-derived MSCs [56]. Furthermore, this study also demonstrated increased neo-angiogenesis in endothelial cells after exposing them to the supernatant of adipose tissue-derived MSCs. These discrepancies in terms of the pro- versus anti-tumorigenic effects of MSCs may be related to differences in MSC donors and sources as well as in harvesting and culture techniques [57].

In our study, not only two-dimensional but also three-dimensional co-culture models were used. There are several advantages of three-dimensional co-culture systems including a higher degree of structural complexity [58]. The applied conical agarose microwell arrays offer further advantages, namely, the ability to examine a plethora of replicates per treatment condition and to study the co-cultures over a long period of several weeks [59,60]. In contrast to previous experiments, in which direct breast cancer-MSC co-culture exhibited both increased growth rates and absolute number of spheroids compared to breast cancer mono-cultures, we could not detect differences in terms of the aggregated spheroid volumes between HNSCC-MSC co-cultures and HNSCC mono-cultures [59].

MSC co-cultures may theoretically be able to alter the number of radiation-induced DNA double-strand breaks in HNSCCs, as the expression of extracellular matrix receptors (e.g., β1-integrin), HNSCCs’ proliferation rates and cell cycle distribution, as well as cytokine levels (e.g., interleukin-6, TGF-β), all factors that have been shown to influence DNA damage repair after irradiation [61,62,63], could be altered by MSC co-cultures. However, γH2AX foci analyses could not detect differences in the number of residual DNA double-strand breaks after irradiation with 2 Gy or 8 Gy in UD-SCC-5 and Cal27 cells. Only FaDu cells exhibited slightly higher numbers of residual DNA double-strand breaks after irradiation with 8 Gy, when co-cultured with MSCs. To the best of our knowledge, this is the first study regarding the influence of MSC co-cultures on the DNA damage repair of HNSCCs after irradiation. The relevance of our findings and the exact mechanisms, however, warrant further investigation.

As CTGF plays an important role in cancer initiation and progression of several cancer types and is involved in the interaction between MSCs and tumor cells [64,65,66,67], we analyzed CTGF levels within HNSCC mono-cultures and HNSCC-MSC co-cultures. Similar to our study, MSCs were the main source for CTGF secretion in a co-culture system consisting of tongue cancer cells and MSCs [68]. However, in contrast to our study, the co-culture systems were found to have significantly higher CTGF levels than MSC mono-cultures. Using neutralizing antibodies against TGF-β, the authors could show that TGF-ß produced by the tumor cells was a main regulator of increased CTGF production in MSCs. Differences in the co-culture set-ups and in MSC donors may be potential explanations for this discrepancy. As CTGF is involved in HNSCC proliferation, migration, and invasion [65,68], the absent increase of CTGF levels in HNSCC-MSC co-cultures (notably, in three different HNSCC cell lines) compared to MSC mono-cultures is a relevant finding, even though further validating studies are required. On the other side, CTGF levels were substantially higher in the co-culture systems than in the HNSCC mono-culture system, raising the question regarding the clinical implications of this finding. Considering the fact that CTGF may serve as potential target (as currently studied in pancreatic cancer within a phase III trial: NCT03941093), further studies exploring the relevance of CTGF in the HNSCCs’ tumor microenvironment seem reasonable.

In summary, MSCs were not found to exert radioprotective effects on HPV-negative and HPV-positive HNSCCs in two- and three-dimensional experiments. Neither the irradiation-induced G2/M-arrest nor early and late apoptosis rates of HNSCCs were altered through direct or indirect MSC co-culturing. Even though CTGF levels were considerably higher in HNSCC-MSC co-cultures, there were no differences between these co-cultures and MSC mono-cultures, suggesting no co-culture-mediated increase of CTGF levels. Further studies are needed to validate these findings in vivo in order to proceed with MSC-based treatments for radiotherapy-induced toxicities.

## 4. Materials and Methods

### 4.1. Cells and Cell Culture

Human MSCs were harvested from the bone marrow of two healthy volunteers as described previously [69]. Written consent was obtained prior to isolation, and this investigation was approved by the Heidelberg University Ethics Committee (#S-384/2004) and by the Freiburg University Ethics Committee (#436/20). The minimal MSC-defining criteria as outlined by the International Society of Cell Therapy have been validated previously [69,70]. FaDu and Cal27 cells were purchased from the American Type Culture Collection (ATCC, Manassas VA, USA), Detroit562 was purchased from Cell Lines Service (CLS GmbH, Eppelheim, Germany), and UD-SCC-2, UD-SCC-3, and UD-SCC-5 were provided by A.K. [71]. MSCs were cultured in StemMACS™ Expansion Media (Miltenyi Biotec, Bergisch-Gladbach, Germany). Detroit562, UD-SCC-3, and UD-SCC-5 HNSCC cell lines were cultured in Dulbecco’s Modified Eagle Medium (DMEM, Biochrom, Berlin, Germany), FaDu and Cal27 in Eagle’s Minimum Essential Medium (EMEM, Biochrom), and UD-SCC-2 in RPMI 1640 (Biochrom), all supplemented with 10% bovine serum and 1% penicillin/streptomycin. In order to distinguish HNSCCs from MSCs, HNSCCs were labeled with ZsGreen using the lentiviral vector rLV.EF1.ZsGreen1-9 (Takara Bio Europe, Saint-Germain-en-Laye, France) according to the manufacturer’s instructions. MSC-conditioned medium (MSC-CM) was created by culturing 1 × 10^6^ MSCs/T75 flasks for 48 h within DMEM (for experiments with the HNSCC cell lines Detroit562, UD-SCC-3, and UD-SCC-5), EMEM (for experiments with FaDu and Cal27), or RPMI (for experiments with UD-SCC-2). Direct HNSCC-MSC co-culture experiments were performed with a ratio of 1:1. Cells were incubated in 37 °C and 5% CO_2_, and culture medium was replaced every 3–4 days.

### 4.2. Radiation Treatments

Photon irradiation was conducted at room temperature using a ^137^Cs laboratory irradiator (Gammacell 40 Exactor, MDS Nordion, Ottawa, ON, Canada) with a dose rate of 0.83 Gy/min. Unirradiated control samples were also stored at room temperature for the same time period as the irradiated samples.

### 4.3. Clonogenic Survival

Clonogenic survival assays were used to evaluate potential radioprotective effects of MSCs and MSC-CM on HNSCCs. Between 200 (0 Gy) and 3000 (8 Gy) HNSCCs were plated in 6-well plates and allowed to attach. The same MSC cell number was added for the direct HNSCC-MSC co-cultures, resulting in a 1:1 ratio. At 24 h, samples were either sham-irradiated or irradiated with 2 Gy, 4 Gy, 6 Gy, or 8 Gy. HNSCCs were either cultured in their respective medium, both as mono-cultures and as direct HNSCC-MSC co-cultures, or cultured in MSC-CM as indirect HNSCC-MSC co-cultures. Colonies were stained with crystal violet/methanol solution at about 2 weeks after treatment, and colonies consisting of 50 or more HNSCCs (MSCs were not included) were counted. Cellular survival fraction was calculated using the following formula: (#HNSCC colonies/#plated HNSCC cells)_treated_/(#HNSCC colonies/#plated HNSCC cells)_untreated_. SER values were determined using the formula: Radiation dose resulting in 10% survival_mono-culture_/radiation dose resulting in 10% survival_co-culture_. Basal plating efficiency in the controls (0 Gy) as shown in Figure S1 was defined as #HNSCC colonies/#plated HNSCCs.

### 4.4. Viability

Cellular viability at 96 h after irradiation was determined using resazurin assays. At 24 h before radiation treatment, 2 × 10^3^ cells/well were plated in 96-well plates and allowed to attach. Cells were incubated for 96 h after irradiation, before 10 µL 0.3 mg/mL resazurin (Sigma, Steinheim, Germany) was added to each well (containing 100 µL culture medium) and incubated for 4 h. Colorimetric analyses were carried out by measuring light absorbance at 570 nm and 600 nm with a VersaMax microplate reader (Molecular Devices, LLC, Sunnyvale, CA, USA). Background absorbance at 600 nm was subtracted from the absorbance at 570 nm.

### 4.5. Proliferation

For proliferation measurements, 1 × 10^4^ cells were plated in 6-well plates and 24 h later irradiated with doses between 2 and 8 Gy. The number of viable cells was assessed at 96 h after irradiation by staining them with 0.4% trypan blue solution (Sigma, Steinheim, Germany) and counting in a Neubauer chamber. The number of viable cells at 96 h after sham-irradiation was calculated both for the HNSCC mono-culture and the HNSCC-MSC co-culture, allowing to calculate the relative proliferation rate at 0 Gy (Figure S2).

### 4.6. Three-Dimensional Survival Assays

Three-dimensional survival was assessed using a standardized conical agarose microwell array as described previously [59]. A cell suspension of either HNSCC mono-culture or HNSCC-MSC co-culture was seeded in 3D CoSeedis™ Chip880 arrays (abc biopply, Solothurn, Switzerland) in a concentration resulting in an average of 10 cells per microwell. At 24 h after seeding, matrices were either sham-irradiated or irradiated with 8 Gy. Culture medium was replaced every 7 days until fixation. Quantitative measurements of the tumor spheroid areas were performed after 3 weeks for FaDu or after 4–8 weeks for UD-SCC-5 and Cal27 using a high-resolution scanner (CanoScan 9000F Mark II, Canon Inc., Tokyo, Japan with the transmitted light modus at 1200 dpi. ImageJ software version 1.52p (National Institutes of Health, Bethesda, MD, USA) was used to measure the projected area of each cell aggregate and to calculate the percentage of remaining aggregates after irradiation compared with non-irradiated controls. Following the initial description of this conical agarose microwell array, average spheroid volume as well as the sum of all spheroid volumes were calculated [59].

In addition, conical agarose microwell arrays were fixed with 2% paraformaldehyde solution overnight. Microwells were subsequently sealed with warm 2.4% low-melting point agarose. After paraffin embedding and casting into a block, sections with a thickness of 2 μm were cut and mounted onto slides. Slides were stained with hematoxylin and eosin (H&E) according to institutional routine pathology protocols.

### 4.7. Cell Cycle and Apoptosis Measurements

HNSCCs or HNSCC-MSC co-cultures were plated in T25 flasks and left to attach prior to radiation treatment. At 24 h and 96 h after irradiation, cells were harvested, fixed in 3% paraformaldehyde solution, and permeabilized in ice-cold 70% ethanol. After three washing steps with 0.5% bovine serum albumin solution, samples were incubated with an Alexa647-coupled antibody against cleaved caspase-3 in a 1:20 dilution (BD Pharmingen, Heidelberg, Germany) for 1 h at room temperature. Following centrifugation with 300× *g* for 5 min, specimens were incubated in 1 µg/mL DAPI solution.

In order to differentiate between early and late apoptosis, annexin-V/7-AAD measurements were performed. Cells were harvested at 96 h after irradiation with 8 Gy and stained using the PE Annexin-V Apoptosis Detection Kit with 7-AAD (BioLegend, London, UK) following the manufacturer’s instruction.

Flow cytometry measurements of cell cycle and apoptosis were performed using a BD FACSVerse™, (BD Biosciences, San Jose, CA, USA) while the following analyses were conducted using FlowJo™ v10 software (FlowJo, Treestar Inc., Ashland, OR, USA). As HNSCCs were labelled with ZsGreen, ZsGreen-negative MSCs could be excluded from the analyses.

### 4.8. DNA Double-Strand Break Repair

γH2AX immunofluorescence stainings were used to determine the number of residual DNA double-strand breaks after irradiation. For each well of a 24-well plate, 2 × 10^4^ HNSCCs, either alone or co-cultured with the same number of MSCs, were plated and irradiated with 2 Gy or 8 Gy. After 24 h, cells were fixed with 4% paraformaldehyde solution and permeabilized using ice-cold ethanol (70%). After three washing steps with 0.5% bovine serum albumin solution, cells were incubated with a monoclonal antibody against γH2AX (Ser139) (1:100 dilution, mouse IgG1, κ, Biolegend) overnight at 4 °C. Afterwards, cells were incubated with an Alexa Fluor™-594-coupled antibody (1:250 dilution, goat anti-mouse, IgG, Invitrogen, Darmstadt, Germany) for 90 min, before nuclei were counterstained with DAPI for 5 min. From each well, 10 images were acquired at 20× magnification using an Olympus IX51 microscope (Olympus Corporation, Tokyo, Japan) and combined to one large image (without stitching). Own software algorithms developed by R.L.P. in MATLAB software version R2017b (MathWorks, Natick, MA, USA) were applied to automatically count γH2AX foci in HNSCC nuclei. Local thresholding was applied to detect the nuclei in the DAPI channel. Closely spaced nuclei were separated using watershed segmentation and nuclei touching the edges of any tile image were excluded. The ZsGreen channel was used to exclude MSCs and remaining HNSCC nuclei were filtered for size, aspect ratio, circularity, compactness, maximum concavity depth, and a concavity-based measure of contour irregularity to exclude artefacts. For each nucleus, foci were detected in the γH2AX channel using a seed segmentation approach after background subtraction. Watershed segmentation was applied to separate closely spaced foci. The foci were then filtered by size, local signal-to-noise ratio, and average intensity in the original image to exclude artefacts. For each treatment condition, foci of three replicates with typically 200–300 (up to >1000) nuclei per individual sample were counted.

### 4.9. Enzyme-Linked Immunosorbent Assays

As CTGF plays a vital role in cancer initiation and progression, is involved in the interaction between MSCs and tumor cells, and has emerged as a potential mitigator of the cellular radiation response, the secretion of CTGF in relation to the presence of MSCs was investigated in HNSCC cultures [64,65,66,67]. Either 5 × 10^4^ HNSCCs/well (mono-culture) or a 1:1 ratio of 5 × 10^4^ HNSCCs and 5 × 10^4^ MSCs/well (direct co-culture) or 5 × 10^4^ HNSCCs/well cultured in MSC-CM (indirect co-culture), were seeded in 12-well-plates and irradiated after 24 h. At 24 h after irradiation, supernatants were harvested and stored at −20 °C. CTGF concentration was measured using the Human CTGF SimpleStep^®^ ELISA Kit (Abcam, Cambridge, UK) according to manufacturer’s instructions. Absorbance was measured at 450 nm with a VersaMax microplate reader (Molecular Devices, LLC, Sunnyvale, CA, USA). The standard curve was created using GraphPad Prism 9 software (GraphPad Software Inc., San Diego, CA, USA). In order to distinguish secretion of HNSCCs from secretion of MSCs, several control samples (MSCs alone, MSC-CM with the respective culture media of the according HNSCC cell line) were measured in terms of CTGF levels, too.

### 4.10. Statistics

At least three replicates were used for all experiments. Values are presented as mean with standard deviations. Clonogenic survival curves were modeled using Sigma Plot version 13 (SyStat Software, San Jose, CA, USA) according to the linear-quadratic model. Potential differences between survival groups were examined using paired Student’s *t*-tests (for the entire survival curve) or one-way ANOVA with post-hoc Dunnett’s tests (for the survival rate after 8 Gy). In general, statistical tests were only performed regarding differences between HNSCC mono-cultures and HNSCC-MSC co-cultures. As there were more than two groups for these comparisons, one-way ANOVA with post-hoc Dunnett’s tests were carried out. GraphPad Prism 9 software was used for the statistical analyses and visualization of the results. *p* ≤ 0.05 was considered statistically significant throughout the study.

## Data Availability

The data presented in this study are available on request from the corresponding author.

## Supplementary Materials

The following supporting information can be downloaded at: https://www.mdpi.com/article/10.3390/ijms23147689/s1.
