# Supplementary material for: Human Mesenchymal Stromal Cells Do Not Cause Radioprotection of Head-and-Neck Squamous Cell Carcinoma
Source: Int J Mol Sci. 2022 Jul 12;23(14):7689. doi: 10.3390/ijms23147689 (PMC9323822; doi:10.3390/ijms23147689)
Supplement: Supplementary file 1 [file ijms-23-07689-s001.zip › Supplementary Table S1.pdf]

Supplementary Table S1. Sensitizer Enhancement Ratio (SER) values calculated at 10% survival in the clonogenic survival assays. Radiation doses resulting in 10% survival were calculated based on the fitted survival curves according to the linear-quadratic model. SER values were computed with the formula: Radiation dose at 10% $survival_{mono\text{-}culture}$/Radiation dose at 10% $survival_{co\text{-}culture}$. No statistical analyses were performed regarding the SER values, as the different groupswere compared with paired t-tests (for the entire curves) or ANOVA with post-hoc Dunnett's tests (for the 8 Gy dose).

	**UD-SCC-5**	**FaDu**	**Cal27**	**UD-SCC-3**	**Detroit562**	**UD-SCC-2**
**MSC1**	1.13	0.90	0.92	0.95	1.10	1.08
**MSC2**	1.10	0.93	0.89	0.88	1.10	1.09
**MSC1-CM**	1.28	0.97	0.85	1.06	1.30	1.19
**MSC2-CM**	1.17	0.94	0.95	1.00	1.19	1.21
