# Supplementary figures and images for: Human Mesenchymal Stromal Cells Do Not Cause Radioprotection of Head-and-Neck Squamous Cell Carcinoma
Source: Int J Mol Sci. 2022 Jul 12;23(14):7689. doi: 10.3390/ijms23147689 (PMC9323822; doi:10.3390/ijms23147689)

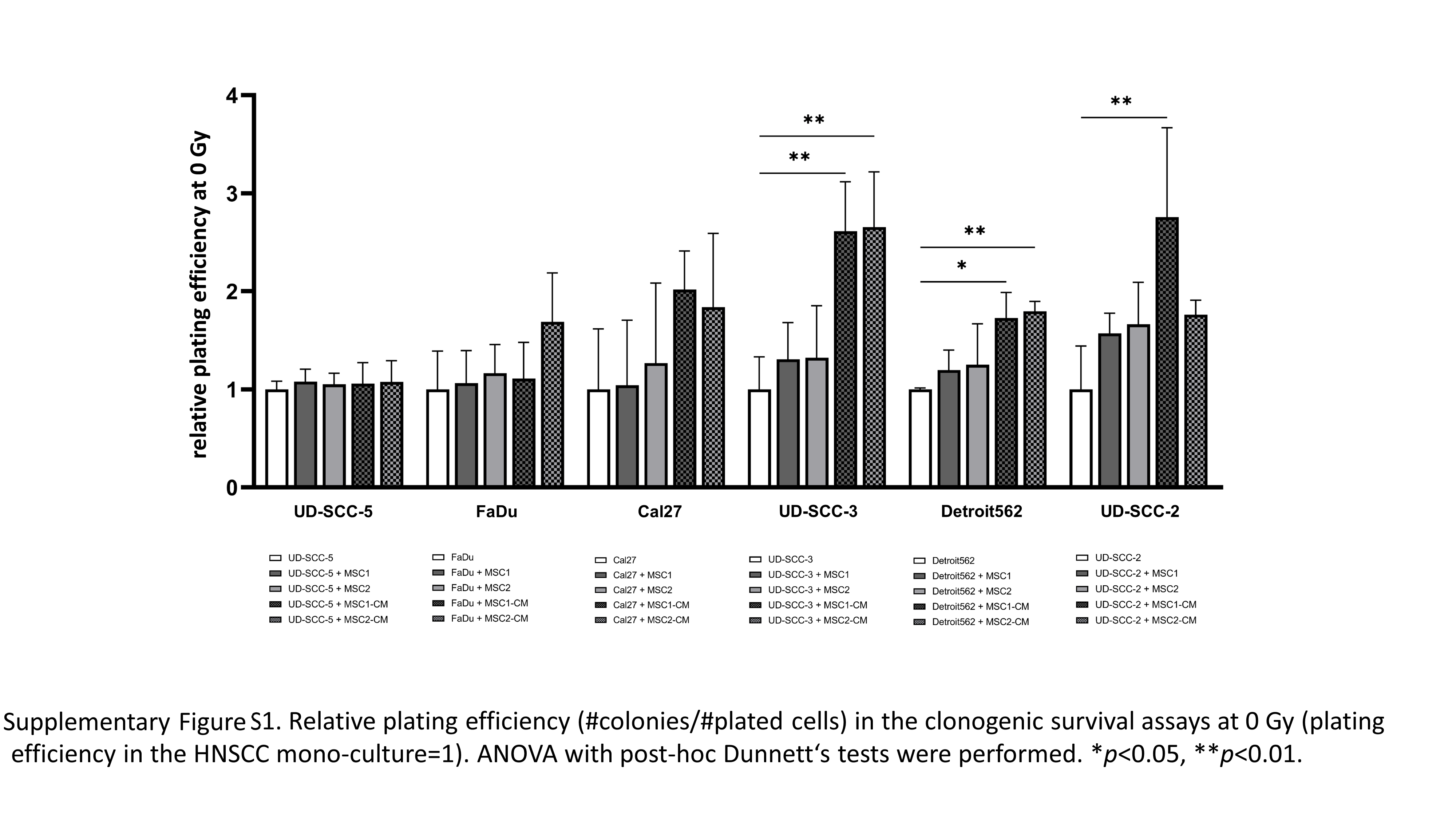

Supplement: Supplementary file 1 [file ijms-23-07689-s001.zip › Supplementary Figure S1.tif]

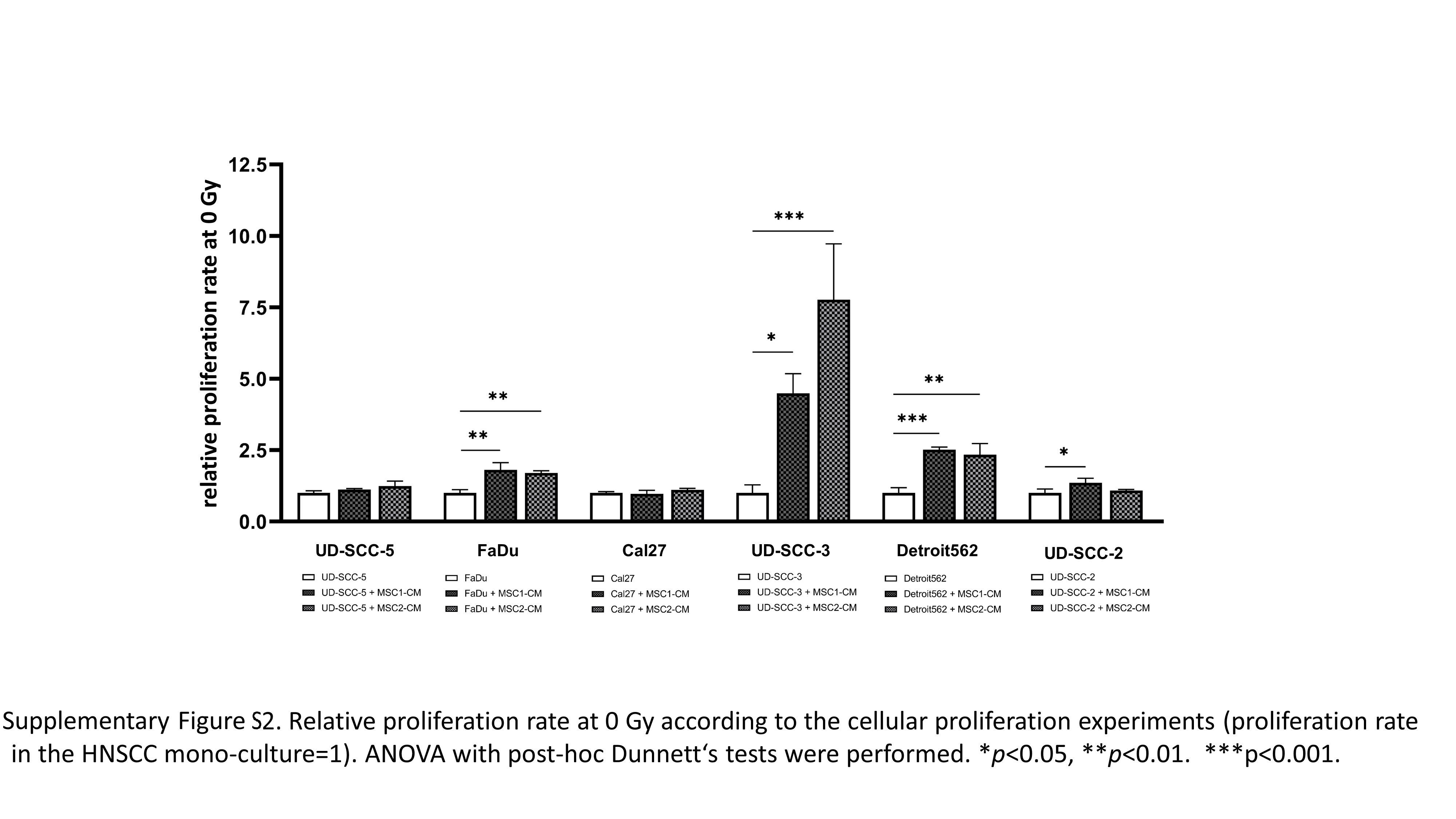

Supplement: Supplementary file 1 [file ijms-23-07689-s001.zip › Supplementary Figure S2.tif]

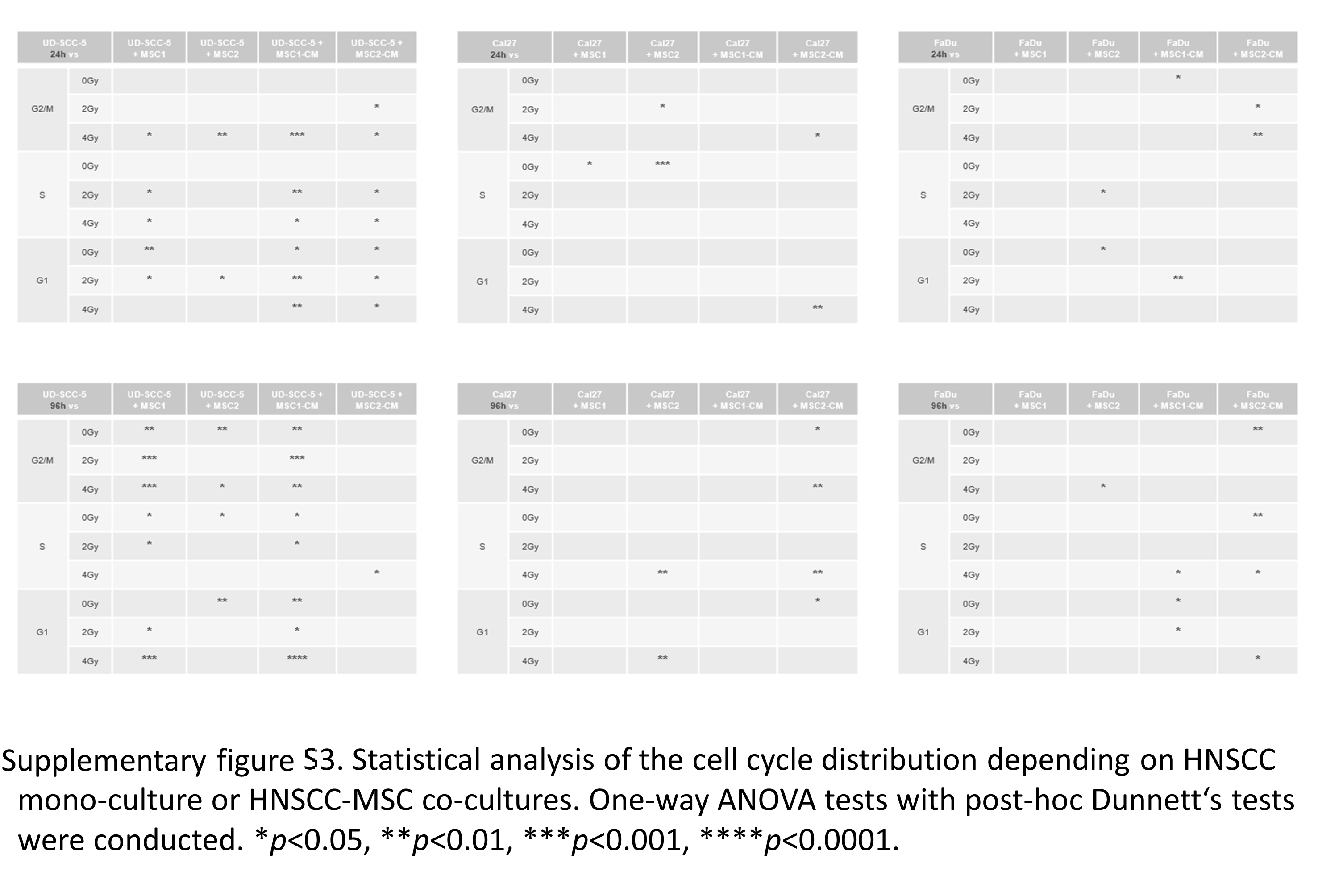

Supplement: Supplementary file 1 [file ijms-23-07689-s001.zip › Supplementary Figure S3.tif]

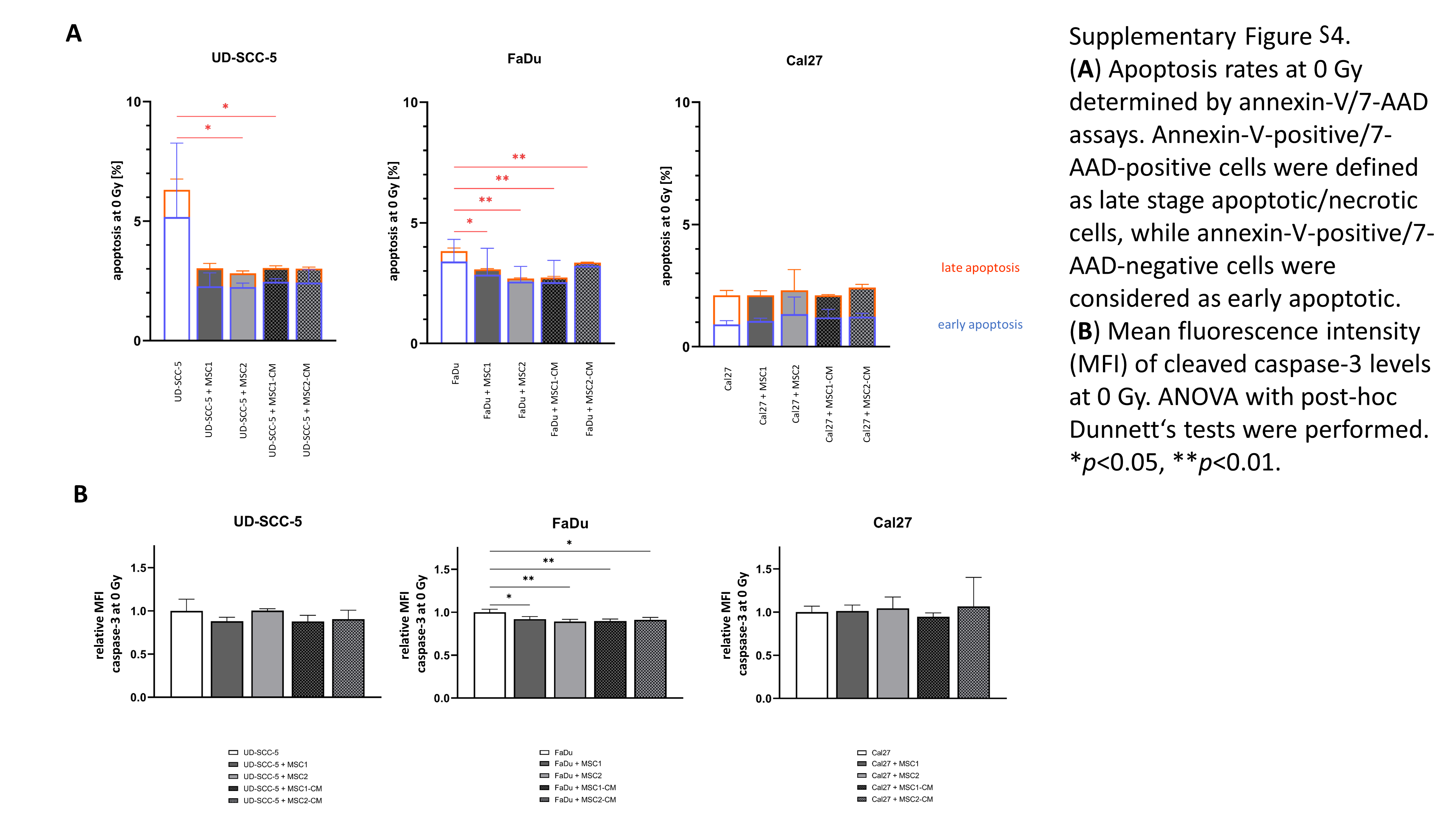

Supplement: Supplementary file 1 [file ijms-23-07689-s001.zip › Supplementary Figure S4.tif]
